# Low Evidence for Tinnitus Risk Factors: A Systematic Review and Meta-analysis

**DOI:** 10.1007/s10162-022-00874-y

**Published:** 2022-11-15

**Authors:** Roshni Biswas, Eleni Genitsaridi, Natalia Trpchevska, Alessandra Lugo, Winfried Schlee, Christopher R. Cederroth, Silvano Gallus, Deborah A. Hall

**Affiliations:** 1 Hearing Sciences, School of Medicine, Mental Health and Clinical Neurosciences, University of Nottingham, Nottingham, UK; 2 Department of Environmental Health Sciences, Istituto di Ricerche Farmacologiche Mario Negri IRCCS, Milan, Italy; 3 NIHR Nottingham Biomedical Research Centre, Nottingham, UK; 4 Department of Physiology and Pharmacology, Karolinska Institutet, Stockholm, Sweden; 5 Department of Psychiatry and Psychotherapy, University of Regensburg, Regensburg, Germany; 6 School of Social Sciences, Heriot-Watt University Malaysia, Putrajaya, Malaysia

**Keywords:** Exposures, Epidemiology, Risk factors, Case–control, Cohort, Tinnitus

## Abstract

**Aims/Hypothesis:**

Identifying risk factors for tinnitus could facilitate not only the recommendations for prevention measures, but also identifying potential pathways for new interventions. This study reports the first comprehensive systematic review of analytical observational studies able to provide information about causality (i.e., case–control and cohort designs).

**Methods:**

A literature search of four electronic databases identified epidemiological studies published on tinnitus and different exposures. Independent raters screened all studies, extracted data, and evaluated study quality using the Newcastle–Ottawa Scale. Reported relative risks (RR), hazard ratios (HR), odds ratios (OR), and prevalence ratios (PR) with 95% confidence intervals (CI) were used to compute crude estimates of RR for tinnitus risk factors.

**Results:**

From 2389 records identified, a total of 374 articles were read as full text (24 reviews, 301 cross-sectional studies, 42 cohort studies, and 7 case–control studies). However, from 49 case–control and cohort studies, only 25 adequately reported risk ratios. Using the findings from these studies, positive causal associations were found for various hearing-related factors (i.e., unspecified hearing loss, sensorineural hearing loss, occupational noise exposure, ototoxic platinum therapy, and otitis media). Evidence was also found for a number of non-otological risk factors including temporo-mandibular joint disorder, depression, chronic obstructive pulmonary disease, and hyperlipidemia. Negative associations indicating preventative effects were found for diabetes and high alcohol consumption. No associations were found for low alcohol consumption, body mass index, head injury, heart failure, hypertension, leisure noise exposure, migraine, rheumatoid arthritis, sex, smoking, stroke, and whiplash. However, with the exception of unspecified hearing loss, these findings resulted from pooling no more than 4 studies, illustrating that the vast majority of the associations still remain inconclusive.

**Conclusions:**

These systematic review and meta-analysis confirm a number of otological and non-otological risk factors for tinnitus. By highlighting major gaps in knowledge, our synthesis can help provide direction for future research that will shed light on the pathophysiology, improve management strategies, and inform more effective preventions.

**Supplementary Information:**

The online version contains supplementary material available at 10.1007/s10162-022-00874-y.

## Introduction

Subjective tinnitus is a common condition among adults with a point prevalence of almost 15%, as recently measured in Europe [1]. While recent years have seen remarkable progress in understanding tinnitus heterogeneity [2], the risk factors for tinnitus as well as the mechanisms of tinnitus generation and maintenance are not well understood [3]. This gap in knowledge is considered one of the major roadblocks in the pathway to tinnitus cure. Thus, identifying and quantifying the relationship between tinnitus and related exposures (risks) could shed some light on underlying pathophysiology, improve management strategies, and inform preventive interventions [3, 4].

The most widely reported risk factor for tinnitus is hearing loss [3]. Environmental influences that damage the auditory system and lead to hearing loss, such as the exposure to loud noise and ototoxic medications, can also trigger tinnitus [5]. A wide number of non-otologic risk factors have been described, but variability in study methodology and quality makes synthesis of findings challenging, and a systematic review of these factors has managed to provide only a narrative synthesis of study results [6]. Potential non-otologic factors include whiplash and neck trauma, blast-injury and traumatic brain injury, and stress [5, 7]. Neurological conditions like tension-type headaches, physical conditions such as temporomandibular joint disorders, and audiological conditions like hyperacusis have been related with tinnitus [8–10], but the direction of these relationships remains rather uncertain. Lifestyle factors like diet, smoking, alcohol consumption, hypertension, and obesity have also been hypothesized to be related to tinnitus [3, 11]. Studies in twins, adoptees, and familial aggregation have also suggested that tinnitus runs in families due to genetics [12–14], something that has recently been confirmed in genome-wide association and whole genome sequencing studies including replication cohorts [15, 16]. In 2012, patients and professionals prioritized unmet research questions which included uncertainties around the role of dietary factors, electromagnetic energy waves, sex hormones, and allergies in tinnitus [17]. It is unclear whether that call has driven the research agenda in these directions.

Since published evidence on exposures is primarily from cross-sectional studies, it is difficult to conclude if the relationship between the exposure and tinnitus is correlational or causal [6, 18]. Moreover, most exposures have been assessed either in specific population groups such as patients attending hearing or tinnitus clinics or from specific geographical areas or demographic characteristics. For example, the epidemiology of hearing loss study provides valuable information on tinnitus. However, the study population is limited to older adults who are residents of the township of Beaver Dam, Wisconsin, USA [19, 20]. This information lacks generalizability as risk factors do not necessarily affect all population groups in the same way [21, 22]. For conditions in which aging is a known risk factor, risks in older adults are different from the risk in the general population which reflects the effects across the life span. Difficulties also arise when the underlying biological mechanism of interaction between an exposure and outcome is complicated and unclear. For example, studies have found people with normal audiometric threshold having self-reported hearing difficulty and vice versa [23–25]. These complexities preclude finding a straightforward association.

From a methodological point of view, to infer causality, exposure to the variable of interest should ideally occur before the onset of tinnitus symptoms, or before an intervention to alleviate tinnitus symptoms or before a prevention strategy to avert tinnitus symptoms. Unfortunately, most tinnitus-related studies measure the risk factor and tinnitus simultaneously in cross-sectional designs and this provides the lowest level of evidence for causal inference [6, 26]. Instead, analytical observational studies (i.e., case–control and cohort designs) are required for the highest level of evidence for causal inference. Through the identification of cross-sectional, case–control, and cohort studies of tinnitus which investigated any potentially relevant exposures, the present study aims (i) to identify which exposures have been reported by analytical observational studies and hence have a high level of evidence for causal inference and (ii) to determine the strength of evidence for those exposures using meta-analysis.

## Methods

A systematic literature review was conducted to identify all publications providing information on the relationship between various exposures and any tinnitus. The review protocol was pre-registered in PROSPERO [27]. Reporting follows the meta-analysis of observational studies in epidemiology (MOOSE) guidelines (Supplementary Material 1). The systematic review did not require ethics committee or Institutional Review Board approval since human subjects per se were not studied, and the data reviewed are in the public domain.

### Search Strategy

The initial search string was developed for MEDLINE using a combination of Medical Subjects Headings (MeSH) and text words related to tinnitus, related exposures, and prevalence. The common terminologies were identified by reviewing MeSH terms and keywords used in MEDLINE to describe a pre-defined set of key publications. Different versions of these syntax were piloted to design the final search string that successfully recovered all of the pre-defined articles. The final MEDLINE search string was adapted accordingly to fit the other medical/healthcare databases used in our search, namely Embase, Cochrane Database of Systematic Reviews (CDSR), and Cumulated Index to Nursing and Allied Health Literature (CINAHL) (Table 1). Multiple databases were searched in an effort to identify all available studies and all authors contributed to the development of the search syntax, with AL, SG, CC, and DAH having conducted previous systematic reviews. The literature search was conducted to identify all studies published on tinnitus and potential risk factors on 29/11/2017 and updated on 06/11/2019. No restriction was applied on the start date.

**Table 1:** Search syntax for each database

**Name of database**	**Search string used**
CDSR	tinnitus:ti (among Cochrane Reviews)
CINAHL (Cumulated Index to Nursing and Allied Health Literature)	MH tinnitus AND “risk factor**” OR epidemiol* OR cohort OR “case control” OR “cross sectional” OR survey* OR longitudinal OR “pooled analysis" OR “meta analysis” OR representative*
Embase	Tinnitus:ti,ab,kw AND (prevalence:kw OR incidence:kw OR “risk factors” OR case–control:ti,ab OR cohort,ti:ab,kw OR cross-sectional:ti,ab,kw OR meta-analysis:ti,ab OR pooled-analysis:ti,ab OR survey:ti,ab OR representative:ti,ab OR longitudinal:ti,ab) AND [embase]/lim NOT [MEDLINE]/lim AND (“article”/it OR “article in press”/it OR “review”/it)
MEDLINE (Pubmed)	(“tinnitus” [MeSH Terms] OR “tinnitus”[All Fields]) AND (“prevalence"[MeSH Terms] OR “incidence” [MeSH Terms] OR prevalence[OT] OR incidence[OT] OR “risk factors” [MeSH Terms] OR “risk factors” [All fields] OR “case–control” [Tiab] OR “cohort” [Tiab] OR “cohort” [OT] OR “cross-sectional” [Tiab] OR “cross-sectional” [OT] OR meta-analysis[tiab] OR pooled-analysis[tiab] OR survey[tiab] OR representative[tiab] OR longitudinal[tiab])

*CDSR* Cochrane Database of Systematic Reviews

Studies were included in the present review if they satisfied the eligibility criteria mentioned in the following sections and were published as a full text in English language. The team did not have adequate resources to translate articles that are not published in English.

### Eligibility Criteria

#### Study Design 

Eligible study designs included cross-sectional (including population-based surveys on specific sub-populations), case–control or cohort studies, meta-analyses, pooled-analyses, and systematic reviews. Case reports, case series, letters to the editor, book chapters, conference proceedings, dissertations, and these were excluded. No restrictions in settings were applied.

#### Study Population 

Human subjects providing data on tinnitus (including pulsatile tinnitus) and associated factors and population-based tinnitus surveys were included. Animal studies were excluded. No restrictions in participant age were applied.

#### Exposures 

All potential exposures related to tinnitus were considered, such as socio-economic characteristics (e.g., level of education), lifestyle habits (including tobacco smoking, alcohol drinking, dietary patterns, obesity, physical activity, mobile use), comorbidities (including depression, anxiety, vertigo, selected cardiovascular diseases, history of trauma), any medications (e.g., aspirin, ototoxic drugs), and otologic conditions. Studies assessing treatment efficacy for tinnitus therapies and with concomitant medical conditions like Meniere’s Disease were excluded.

#### Comparators/Controls 

Subjects not exposed to tinnitus-associated factors were treated as comparators. Relative risks (RR), hazard ratios (HR), odds ratios (OR), and prevalence ratios (PR) were used as the measures of effect to ascertain the excess risk of tinnitus incidence or prevalence for subjects exposed to specific risk factors as compared to those who were not exposed. Studies without a clear comparator group were excluded.

#### Outcomes 

All studies reported information on either tinnitus incidence or prevalence, or some measure of effect for tinnitus-related exposures; namely RR, HR, OR, or PR with 95% confidence intervals (CI).

### Article Screening 

An EndNote library was populated with all articles retrieved from the electronic database search and this library was updated to include the additional articles retrieved from the updated search. Duplicate records were deleted leaving a total of 2389 publications. No further searches (e.g., hand searches) were performed. A stepwise screening method was performed. Any discrepancies were resolved by discussion, with an arbitration provided by a third member of the team.

Titles and abstract screening was conducted independently by two of the authors (RB, EG, NT, and AL). The publications were scored 1–5 where 1, publication not pertinent or of limited interest for our review; 2, publication probably not pertinent or of limited interest; 3, not possible to evaluate on the basis of title/abstract/keywords, only; 4, publication probably pertinent or of interest; and 5, publication pertinent or of clear interest for our review. The two reviewer scores were summed to give total scores ranging between 2 and 10. Only those publications with a combined score of 5 or more were passed to the next screening step.

Full-text screening of potentially eligible studies was again conducted independently by two reviewers (RB, EG, NT). Of the 847 articles eligible for full-text screening, 15 were excluded because the full text could not be obtained and 392 were excluded because they did not fit the eligibility criteria or did not report relevant information. A further 81 articles reported data only on the prevalence or incidence of tinnitus without considering risk factors. This left a total of 374 articles for data extraction, but only 49 of those were analytical observational studies. Some of these referred to the same study, published in multiple articles.

### Data Extraction 

A data extraction form in MS Excel was used to extract the following information: first author, journal, year of publication, country, type of study, name of source database (if any), tinnitus definition, adjustments in the statistical modeling, reporting of prevalence and incidence, followed by the type of exposure assessed and their measure of association (RR, HR, OR, PR, or raw data) (Supplementary Material 2). Data extraction was conducted independently by two of the authors (RB, EG, and NT) and information consolidated through discussion. Authors of included articles were not contacted for further information at this stage.

The included studies used three operational definitions of tinnitus; self-report of “any tinnitus” (AT), self-report of tinnitus that is moderately severe or causes problems getting to sleep (SignificantT), and professionally diagnosed tinnitus (MedT). Given the paucity of analytical observational studies, this variability in tinnitus definition was not taken into account when pooling data for meta-analysis, but the pooled estimates are also reported separately for greater transparency. Pooling was deemed acceptable since we do not interpret specific causal inference from the meta-analysis results. They are primarily to summarize the exposure data.

For summarizing the data, there is currently no recommended classification system for tinnitus-related exposures. Baguley et al. [3] suggested a taxonomy for putative tinnitus risk factors that primarily focused on ototoxic medications and other comorbid conditions related to tinnitus. We expanded these categories to accommodate other tinnitus-related exposures identified through the literature search. This resulted in a taxonomy comprising six domains each with categories and sub-categories. The six domains were (i) socio-demographic, (ii) hearing related, (iii) otological, (iv) potentially ototoxic medications, (v) lifestyle, (vi) comorbidities, and (v) other (Table 2). The data extraction form was expanded iteratively to include the additional exposure categories as they emerged during the data extraction process.

**Table 2:** Taxonomy of tinnitus-related exposures in six main broad domains (bold)

**Category**	**Sub-category**	**Illustrative examples**
**Hearing related**		
Types of measurement	Clinically measured	Hearing loss (unspecified), sensorineural hearing loss
	Self-reported	Hearing difficulty, hyperacusis
Noise exposure	Noise	Occupational and leisure noise
Otologic	Infectious	Otitis externa, otitis media
	Labyrinthine	Cochlear implants, vestibular dysfunction
	Neoplastic	Acoustic neuroma, Schwannoma
	Other	Cerumen, otosclerosis, presbycusis
Ototoxic medications	Antibiotics	Erythromycin, Macrolides
	Anti-neoplastic	Platinum-based therapy
	Other drugs	Nonsteroidal anti-inflammatory drugs
**Lifestyle**		
Lifestyle exposures	Chemical	Chemical solvents
	Physical/other	Computer use, electromagnetic field
Nutrition	Anthropometric	Body mass index, waist to hip ratio
	Diet and nutrition	Dietary patterns, deficiency
	Physical activity	Hours of exercise
Substance use	Alcohol	Drinking status, consumption
	Coffee	Drinking status
	Drug addictions	Drug abuse
	Smoking	Current, exposure to second-hand smoke
**Socio-demographic**		
Age		
Family history		
Marital status		
Race/ethnicity		
Region		Geographic region, urbanization level
Socioeconomic status	Education	School years, education level
	Employment status	
	Income	Yearly income, income level
Sex		
Social environment		Family situation, working conditions
Other demographics	Benefits	Disability benefits, health insurance
	Deprivation score	Deprivation index
**Comorbidities**		
Cardiovascular		Heart failure, hypertension, stroke
Endocrine and metabolic		Diabetes, hyperlipidemia, thyroid disorders
ENT, other	Nasal disorders	Rhinitis, sinusitis
Hepatological		Transplant, cirrhosis, hepatitis B
Immune-mediated		Systemic lupus erythematosus
		Rheumatoid arthritis
Infections (systemic)		Human immunodeficiency virus, Human T-cell lymphotropic virus type 1
Musculoskeletal		Osteoporosis
Neurological		Headache, meningitis, migraine
Neoplastic		Cancer survivors, childhood cancer
Orofacial	Dental conditions	Teeth grinding, clenching
	Musculoskeletal	Temporomandibular joint disorder
Mental		Anxiety, dementia, depression, stress
Renal		Chronic kidney disease
Respiratory		Chronic obstructive pulmonary disease
Sleep disorder		Hypersomnia, insomnia, sleep apnea,
Traumatic	Ear injury	Tympanic membrane perforation
	Head and neck injury	Traumatic brain injury, Whiplash
	Other bodily injury	Bodily injury score
Other systemic conditions		Allergy, chronic illness, disability, menstruation, pregnancy
**Treatments**		
Radiotherapy		Radiation (gamma)
**Other**		
Genetics		Genotype, specific alleles

*ENT* ear, nose and throat; *COPD* chronic obstructive pulmonary disease

### Quality Assessment

All included articles were evaluated for study quality. Our first-level thresholds for quality required the study to have a case–control or cohort study design, as well as to report the measures of effect as risk ratios (e.g., RR, HR, OR, or PR). Analytical observational study designs (i.e., case–control and cohort studies) passed the quality threshold since their findings provide information on likely causal inference as well as the degree of association. Report of risk ratios was also a requirement since these estimates can then be pooled in a data synthesis. Although crude estimates of risk ratios can be calculated from studies providing raw data (numbers and proportions) for exposures and tinnitus, this does not give an unbiased picture as there is risk of confounding and so where raw data was reported, it was not analyzed further.

Those studies passing the first-level quality benchmark were then subjected to a further quality appraisal using the Newcastle Ottawa Scale (NOS). This risk of bias assessment tool for observational studies is recommended by the Cochrane Collaboration [28]. The NOS assigns up to a maximum of nine points for the least risk of bias in three domains: (1) selection of study groups (four points), (2) comparability of groups (two points), and (3) ascertainment of exposure and outcomes (three points) for case–control and cohort studies, respectively. Scores in domain (3) were calculated separately for each exposure, depending on how that exposure had been measured. A total score of zero indicates very low quality and nine very high quality, but there is no explicit cutoff to distinguish high from low quality.

### Data Synthesis

For most exposures, very few studies met the first-level quality thresholds. Exposures with evidence from two or more studies were identified. For each such tinnitus-associated factor, meta-analyses were conducted to quantify the difference in risk in individuals exposed to the factor when compared to the unexposed comparator. Heterogeneity between studies was assessed using the Q and I^2^ tests. The pooled RRs from random-effects models using the DerSimonian and Laird moment estimator of the between-study variance component was evaluated. All statistical analyses were conducted with R (version 3.5.2) software.

In some cases, the published reports were derived from the same study data during the same period and so this paragraph describes how these duplications were handled in the meta-analyses. Two out of the six articles on unspecified hearing loss came from the Epidemiology of Hearing Loss Study; one 5-year follow-up [20] and one 10-year follow-up [29]. Since the individuals followed up for 5 years were included in the 10-year analyses, the most recently reported data was used for the meta-analysis. For the remaining four articles on hearing loss, two came from the Blue Mountains Hearing Study and two from the Taiwan National Health Insurance Resource Database, again from the same study period. Meta-analysis therefore used data reported by Gopinath et al. [30] and Lee et al. [31], respectively. Two out of the three articles on sensorineural hearing loss came from the Taiwan National Health Insurance Resource Database from the same study period. Therefore, the one with the complete information was used [32]. Risk factor information for hypertension, diabetes, and stroke was available from Taiwan National Health Insurance Resource Database for two articles reporting the same study period [33, 34]. Since Chen et al. [33] analyzed information for women and Shih et al. [34] for both sexes, the latter was entered into the meta-analyses.

Data from a number of other studies did not contribute to the meta-analyses because the reference group was not appropriate. The prospective cohort study by Aarhus et al. [35] compared adults with a history of childhood hearing loss to a reference group of adults with normal childhood hearing, and so risk ratios for sensorineural hearing loss and otitis media were not considered. Additional otitis media risk ratios were also omitted as they came from adults with chronic suppurative otitis media in childhood compared to a reference group of adults without a history of otitis media [36]. The study by Dougherty et al. [37] compared military personnel wearing hearing protection at the time of injury compared with a reference group without hearing protection, and so their risk ratios on occupational noise exposure were omitted. Full details from these studies are reported in the Supplementary Material 1.

## Results

**Fig. 1:**
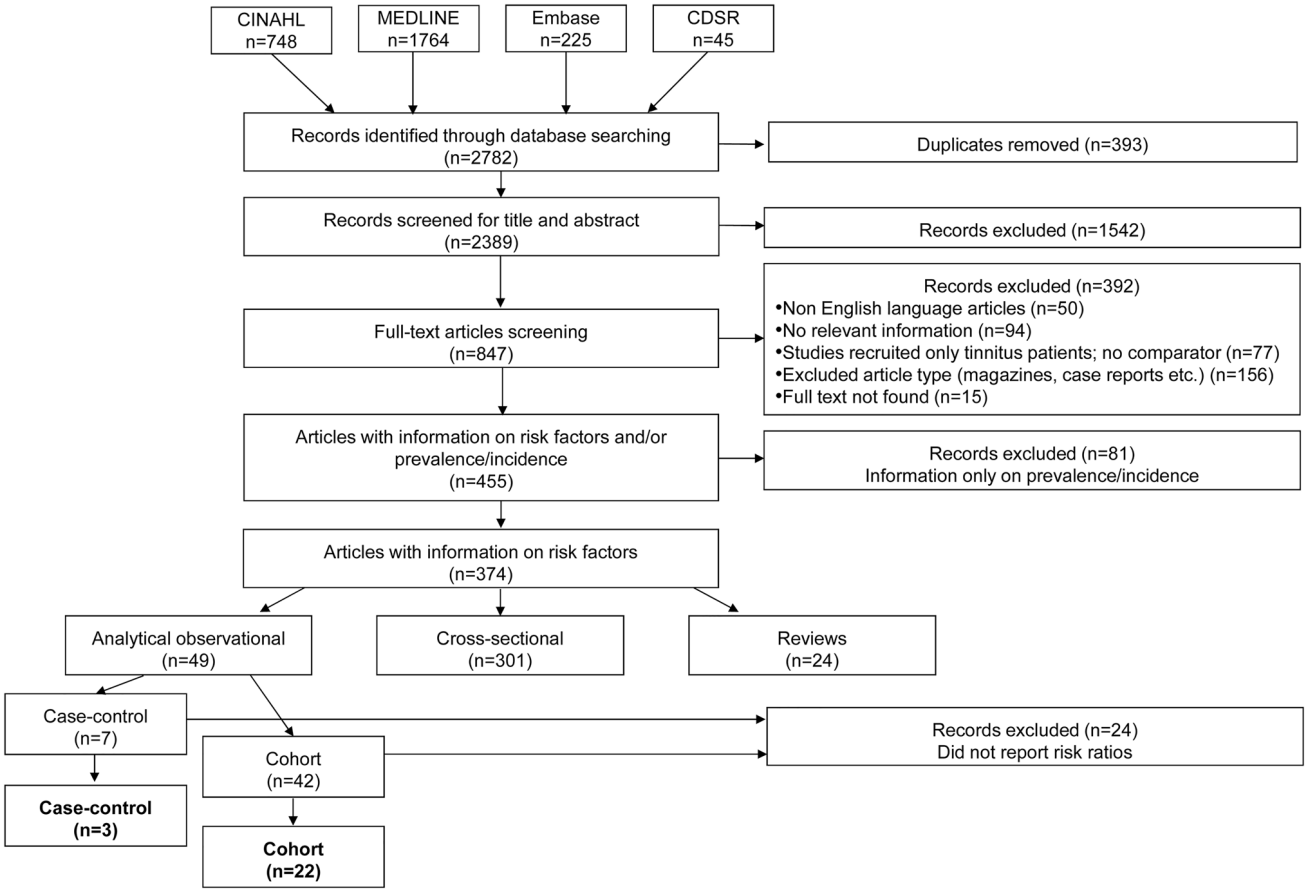
Flow diagram

Of the 374 included studies, 49 were analytical observational studies (7 case–control and 42 cohorts), 301 were cross-sectional studies, and there were 24 reviews (Fig. 1). From those analytical observational studies, only 25 met the first-level quality threshold (i.e., reported risk ratios) and are reported further. Three of the included analytical observational studies evaluated data from case–control studies, and 22 analyzed data from cohort studies. Among the cohort studies, seven were prospective designs, while the rest were from retrospective. All case–control studies were retrospective in design (Supplementary material 1). Table 3 confirms that the average NOS quality assessment scores were at least 6 out of 9 for the exposures analyzed in the meta-analyses. We consider this to indicate high quality.

**Table 3:** Exposures analyzed using meta-analytic approach along with number of high-quality studies and quality assessment scores

**Risk factor**	**Analytical study design**		**Total**	**NOS score mean (SD)**
	**Case–control**	**Cohort**		
***Hearing related***				
**Hearing loss (unspecified)**	**0**	**6**	**6**^**a**^	**8.3 (0.8)**
**Hearing loss (sensorineural)**	**2**	**1**	**3**^**a**^	**7.7 (1.0)**
Leisure noise exposure	1	1	2	7.9 (0.3)
**Occupational noise exposure**	**1**	**2**	**3**^**c**^	**7.1 (0.4)**
**Otitis media**	**2**	**2**	**4**^**b**^	**7.3 (0.7)**
**Platinum (ototoxic)**	**0**	**4**	**4**	**6.0 (1.4)**
***Lifestyle and socio-demographic***				
Alcohol consumption	1	1	2	7.8 (1.0)
Body mass index	1	1	2	8.0 (0.0)
Sex	0	4	4	8.5 (0.5)
Smoking (current)	1	1	2	7.5 (1.0)
***Comorbidities***				
**Depression**	**1**	**1**	**2**	**8.0 (1.4)**
**Diabetes**^b^	**2**	**1**	**3**^**a**^	**8.0 (1.0)**
Heart failure	1	1	2	8.0 (1.0)
Hyperlipidaemia	1	1	2	7.7 (1.2)
Hypertension	1	3	4^a^	7.5 (1.3)
Migraine	1	1	2	7.3 (0.5)
Stroke	1	2	3^a^	8.0 (1.0)
**Temporo-mandibular joint disorder**	**1**	**1**	**2**	**8.3 (1.0)**
Chronic obstructive pulmonary disease	1	1	2	7.5 (0.7)
Rheumatoid arthritis	1	1	2	7.5 (0.7)
Head injury	1	3	4	8.2 (0.8)
Whiplash	1	1	2	7.0 (0.0)

All risk factors in bold indicate a statistically significant association with tinnitus

*COPD*, chronic obstructive pulmonary disease

^a^Not all data from included articles were included in the meta-analyses. See text for details

^b^Negative association with tinnitus

### Description of Included Studies

The 25 analytical observational studies were conducted in Western and Northern Europe, North America (USA), Asia (Taiwan), and Australia. A short description of the 10 prospective cohort studies is given as follows. European studies were from the UK, Germany, and Scandinavia. One UK study evaluated hearing-related information from participants of the Oxford-Family Planning Association contraceptive study — prospective cohort of 17,000 women recruited at 17 clinics in England and Scotland between 1968 and 1974 [38]. One German study was derived from the Study of Health in Pomerania, a population-based prospective cohort study [39]. In Nord-Trøndelag Norway, participants were assessed at 7, 10, or 13 years old during a school hearing investigation and also screened at 20 and 56 years old during Nord-Trøndelag Health Study, specifically for hearing loss from 1996 to 1998 [35, 36]. Another study in Sweden and Finland analyzed the amount of mobile phone use at baseline with self-reported weekly tinnitus [40]. In the USA, one study considered tinnitus data from the Nurses’ Health Study II, a prospective cohort study on 25 to 42-year-old nurses from 14 US states to study conditions and risk factors related to women’s health [41]. Two articles reported follow-up information from the Epidemiology of Hearing Loss Study, a study on adults aged 48–92 years, residing in Beaver Dam, Wisconsin [20, 29]. Finally, in Australia, two studies reported data from the Blue Mountains Hearing Study, a population-based survey of age-related hearing loss in 55–99-year-olds residing in two postcodes of Sydney, Australia [30, 42].

A short description of the 12 retrospective cohort studies is given as follows. European studies were from Germany and Sweden. One study in Sweden assessed preschool teachers’ risks based on retrospectively reported symptom onset [43]. One study in Germany used medical health insurance data for retrospective analyses [44]. In Asia, six articles from Taiwan reported retrospective analyses of medical health insurance data [31–34, 45, 46]. Taiwan’s National Health Insurance system was established in 1995 and covers almost 100% of the population. The database includes records from primary outpatient departments and inpatient hospital care settings and it is maintained and regulated by the Data Science Centre of the Ministry of Health and Welfare of Taiwan. Finally, four studies were from the USA. Two studies reported retrospective analyses of health records of military personnel from military hospital–based databases, namely the Expeditionary Medical Support System, Defense Medical Surveillance System, the Pharmacy Data Transaction Service, and the Theater Medical Data Store [37, 47]. Civilian data comprised two studies reporting the Childhood Cancer Survivor Study dataset. This is a North American multi-institutional collaborative retrospective study of individuals who survived at least 5 years after diagnosis of cancer during childhood or adolescence, and a cohort of siblings not affected by cancer [48, 49].

The three retrospective case–control studies came from a variety of sources. One study again came from the Taiwan National Health Insurance Resource Database [50]﻿. Here, cases comprised patients with medically diagnosed tinnitus and controls matched for age, sex, and year of index date. Another considered tinnitus cases attending an ENT outpatients clinic in an Austrian hospital with ENT controls matched for age, gender, ethnicity, and 3 week index date [51]. The final study examined records from the UK Clinical Practice Research Datalink, which is an anonymized database created in 1987 with ongoing medical records from over 11 million patients provided by approximately 700 general practices [52].

There was a large heterogeneity in how studies have performed their adjustment for important covariates. Of the statistical modeling used, 92% of studies adjusted the risk ratio estimates for age, and 60% for sex/gender. Some studies corrected for neither [32, 50–52]. Hearing loss was used as a covariate in only 16% of studies, despite tinnitus being a primary outcome in 17 of 25 studies.

### Strength of Evidence for Tinnitus Risk Factors 

Cox regression was used as a statistical model in 8 of the included studies. Table 3 reports those exposures reported within more than one included article, for which meta-analysis is possible. In summary, hearing loss [both unspecified (6 studies) and sensorineural (3 studies)], occupational noise exposure (3 studies), otitis media (4 studies), diabetes (3 studies), temporomandibular disorder (2 studies), and ototoxic platinum exposure (4 studies) were the most reliable risk factors for tinnitus.

Supplementary Figs. 1–6 present the forest plots for all hearing-related conditions. Tinnitus was, on average, significantly associated with unspecified hearing loss (RR, 1.94; 95% *CI*, 1.41–2.67; *I*^2^ = 69%; *p* = 0.02; Supplementary Fig. 1), sensorineural hearing loss (RR, 3.68; 95% *CI*, 2.93–7.04; *I*^2^ = 99%; *p* < 0.01; Supplementary Fig. 2), occupational noise exposure (RR, 1.70; 95% *CI*, 1.49–1.94; *I*^2^ = 48%; *p* = 0.17; Supplementary Fig. 3), otitis media (RR, 1.63; 95% *CI*, 1.61–1.65; *I*^2^ = 0%; *p* = 0.77; Supplementary Fig. 4), and platinum therapy (RR, 2.81; 95% *CI*, 1.81–4.36; *I*^2^ = 26%; *p* = 0.25; Supplementary Fig. 5)]. In contrast, leisure noise exposure was not associated with developing tinnitus (RR, 1.36; 95% *CI*, 0.70–2.62; *I*^2^ = 55%; *p* = 0.14; Supplementary Fig. 6).

Of the lifestyle or socio-demographic factors, only high alcohol consumption was negatively associated with tinnitus (RR, 0.94; 95% *CI*, 0.91–0.96; *I*^2^ = 0%; *p* = 0.50; Supplementary Fig. 7) whereas this was not the case for low alcohol consumption (RR, 1.00; 95% *CI*, 0.85–1.19; *I*^2^ = 42%; *p* = 0.19; Supplementary Fig. 8), smoking (RR, 1.15; 95% *CI*, 0.81–1.62; *I*^2^ = 88%; *p* < 0.01; Supplementary Fig. 9), or sex (RR, 1.06; 95% *CI*, 0.92–1.22; *I*^2^ = 77%; *p* = 0.01; Supplementary Fig. 10].

Regarding comorbidities, temporo-mandibular joint disorder was associated with tinnitus (RR, 2.06; 95% *CI*, 1.30–3.27; *I*^2^ = 97%; *p* < 0.01; Supplementary Fig. 11), as well as depression (RR, 1.31; 95% *CI*, 1.28–1.34; *I*^2^ = 0%; *p* = 0.76; Supplementary Fig. 12). In contrast, diabetes (RR, 0.85; 95% *CI*, 0.82–0.88; *I*^2^ = 0%; *p* = 1; Supplementary Fig. 13) was negatively associated with tinnitus.

There was no evidence for an association between tinnitus and the following comorbidities: leisure noise exposure (RR, 1.36, 95% *CI*, 0.70–2.62; *I*^2^ = 55%; *p* = 0.14; Supplementary Fig. 14); body mass index (RR, 0.83; 95% *CI*, 0.62–1.11; *I*^2^ = 78%; *p* = 0.01; Supplementary Fig. 15); heart failure (RR, 0.73; 95% *CI*, 0.44–1.20; *I*^2^ = 90%; *p* < 0.01; Supplementary Fig. 16), hypertension (RR, 0.98; 95% *CI*, 0.96–1.00; *I*^2^ = 0%; *p* = 0.39; Supplementary Fig. 17), stroke (RR, 0.94; 95% *CI*, 0.73–1.21; *I*^2^ = 0%; *p* = 0.69; Supplementary Fig. 18); hyperlipidemia (RR, 1.18; 95% *CI*, 1.00–1.40; *I*^2^ = 28%; *p* = 0.24; Supplementary Fig. 19); rheumathoid arthritis (RR, 1.19; 95% *CI*, 0.97–1.47; *I*^2^ = 67%; *p* = 0.08; Supplementary Fig. 20); migraine (RR, 2.11; 95% *CI*, 0.93–4.79; *I*^2^ = 93%; *p* < 0.01; Supplementary Fig. 21); Chronic obstructive pulmonary disease (COPD) (RR, 0.95; 95% *CI*, 0.90–1.00; *I*^2^ = 0%; *p* = 0.71; Supplementary Fig. 22); head injury (RR, 1.21; 95% *CI*, 0.97–1.52; *I*^2^ = 91%; *p* < 0.01; Supplementary Fig. 23); and whiplash (RR, 1.40; 95% *CI*, 0.95–2.07; *I*^2^ = 60%; *p* = 0.11; Supplementary Fig. 24).

## Discussion

The present study reveals the very limited knowledge on exposures causally related to tinnitus. Using a systematic literature evaluation, 374 articles reported information on exposures related to tinnitus. From this pool, only 13% of articles reported data collected from analytical observational studies (i.e., case–control and cohort studies), of which only half met the quality threshold defined for this review. Thus, from the original set of 374 articles, only 6.7% met our criteria for consideration into the meta-analysis. Our findings confirm the known role of hearing loss in increasing the risk of tinnitus, and this also includes other otological conditions known to affect hearing. Interestingly, our findings also confirm a causal link between temporo-mandibular joint disorder and tinnitus, consistent with previous suggestions [6]. Evidence was also found for a number of other non-otological risk factors including depression, COPD, and hyperlipidemia. Negative associations indicating preventative effects were found for diabetes and high alcohol consumption. These were unexpected findings, but we note that in each case, the pooled estimates come from only two studies and so we have limited confidence that these results are reliable. In the case of diabetes, there is reasonable evidence for a positive association between type 1 and type 2 diabetes and sensorineural hearing loss, and it is suggested that there may be shared risk factors such as glucose processing abnormalities and aging, in addition to some pathologies created by diabetes leading to hearing loss [53]. It is noted that neither of the two included diabetes studies [34, 52] adjusted their models for hearing loss and so this confound needs to be examined in further research. Furthermore, at least one cross-sectional study has suggested an interaction between exposures, such that comorbidity of diabetes and hypertension poses a risk factor for tinnitus [54]. However multi-comorbidities are rarely assessed and are not considered in the current review. This review highlighted little or no evidence for the numerous exposures that have been previously suggested as potential risk factors in previous cross-sectional studies (e.g., migraine, head injury, and whiplash, [6]) or identified as a research priority (e.g., caffeine, [17]).

### Quality and Availability of Evidence

The interpretation of results from systematic reviews depends on the quality of evidence and risk of bias [55]. The degree of reliability of analytical observational studies can be from excellent to very poor depending on selection of study groups, comparability of groups, and ascertainment of exposure and outcomes (three points) for case–control and cohort studies, respectively [28]. Almost half of the cohort studies had to be excluded as they did not report sufficient information, such as risk ratios, and so the quality appraisal was not conducted. However, for those included studies that were evaluated using NOS, the quality assessment score was high. There is an evident need for well-conducted analytical observational studies in the tinnitus field, such that there is enough validity in the results both when studies are considered individually or as a part of a systematic review.

### Gaps in Knowledge

The two most striking gaps highlighted in this review include the lack of analytical observational studies and gaps in knowledge about some known risk factors. It is impossible to elicit the cause-and-effect relationship between potential exposures and tinnitus without analytical observational data. For example, previously Deklerck et al. [6] suggested links between various cardiovascular disorders (e.g., dyslipidaemia, peripheral vascular disease and ischaemic heart disease, and stroke) and tinnitus. However, excluding the cross-sectional studies and taking into account only the high quality studies, our review shows that the current evidence is not reliable. In the case of anxiety disorders that have consistently been related to tinnitus [3, 56], and for which treatments reduce the impact of tinnitus [56], only one case–control study reported a positive association [52]. Similarly, knowledge on the association of otosclerosis and tinnitus results from a single case–control study [20] that has not been replicated elsewhere. Finally, factors like diet and physical activity, for which suggestive evidence of a relationship exists from cross-sectional datasets, have never been investigated in analytical observational studies.

### Self-Reported Tinnitus Versus Medically Diagnosed Tinnitus

Another consideration that arose during the present study is that while most population-based studies rely on self-reports of tinnitus, case–control or retrospective cohorts are often based on electronic health records using medically diagnosed tinnitus (e.g., tagged with an ICD code, International Classification of Diseases) as the outcome of interest. Examples include the Taiwan National Health Insurance Resource Database and Clinical Practice Research Datalink. It is indeed possible to conduct hospital-based case–control studies from ENT clinics or population-based case–control studies from existing healthcare or health insurance databases by selecting tinnitus cases and tinnitus-free controls matched for index dates. However, to obtain accurate results, in both instances, reliable coding of tinnitus is essential. One limitation is that ENT doctors may not reliably record tinnitus codes as this is often a secondary symptom associated with a more primary otological disorder. Another limitation concerns the lack of etiologically meaningful tinnitus subtypes. For example, ICD-10 subcodes for tinnitus specify only left ear, right ear, bilateral, or unspecified. While updates to the ICD codes are possible, this would require global consensus by lead experts in order to have these approved by the World Health Organization and further implemented in national medical systems.

Here, nearly half of the analytical observational studies (13/25) relied on ICD coding from medical or insurance registry data. The forest plots presented in the Supplementary Figures provide pooled estimates for self-reported AT separate from that for MedT and significantT, in addition to the overall pooled estimate. This provides optimal transparency for the reader. Such healthcare or health insurance databases can also be used to conduct retrospective cohort studies. For example, Martinez et al. [57] conducted a retrospective cohort study using UK NHS healthcare records (i.e., Clinical Practice Research Datalink and Hospital Episode Statistics) to determine incidence. Recently, Lugo et al. [58] used medical registry data within the Stockholm Public Health Cohort to establish that women with a previous medical evaluation at the specialty care had lower risk for suicidal attempts. These are convenient methods for hypothesis generation and quantify some degrees of association between an exposure and tinnitus. However, tinnitus in a help-seeking individual is qualitatively different from tinnitus in an individual not seeking medical support, and while the former is evaluated by a healthcare professional, the latter is self-reported [59]. In addition, caution should prevail as individuals that may have been assessed by a physician may suffer less than individuals with self-reported severe tinnitus that have not benefited from medical care. As exemplified by the study from Lugo et al. [58], the relationship between severe tinnitus and suicide attempts may have been underestimated if solely focusing on ICD-coded data. This may be particularly relevant for any psychological or psychiatric oriented study related to tinnitus. Moreover, individuals that have been labeled an ICD code for tinnitus may not necessarily have a severe tinnitus, as it may have been diagnosed as a secondary symptom accompanying for instance hearing loss, or it may have been an acute tinnitus remitting shortly after the auscultation (occasional tinnitus). Similar to other psychiatric traits that cannot be reliably objectively diagnosed, we recommend that a pattern of help-seeking behavior (such as at least one referral to specialty care for a primary tinnitus complaint) may provide increased reliability over primary care diagnoses when performing retrospective studies.

### Limitations

We acknowledge that an inherent limitation of our study is the paucity of risk ratio data available for meta-analysis. The results presented here were conducted on data reported from an average of two or three sources, thereby limiting the generalizability and the validity of the findings. This precludes the possibility of firm causal inferences on the association between tinnitus and exposures. Moreover, the few high-quality studies available have explored a selected set of risk factors with a clear preference for auditory conditions, resulting in major gaps in knowledge for many relevant associated conditions. The latest update was performed on 06/11/2019, and it is possible that new tinnitus results may have been reported since.

Another second limitation is the incapacity to draw conclusion on sex-specific mechanisms, which is acknowledged as being under investigated in the tinnitus field [60]. Whereas we have found no association between tinnitus and sex in the present meta-analysis, a number of studies suggest that there could be influences of sex on, for instance, tinnitus severity. Previous work from Schlee et al. [61] reported greater stress and anxiety in women with constant tinnitus. These findings are consistent with another report showing that women with severe tinnitus have greater odds of suicide attempts, something that is not found in men [58]. It is possible that such mechanisms are, at least in part, due to genetics since woman with tinnitus have almost ten times the risk of having a sibling with tinnitus [λs = 10.25; 95% *CI* (7.14–13.61)] whereas for men, this risk is fivefold [λs = 5.03; 95% *CI* (3.22–7.01)] [12]. A recent cross-sectional study by Basso et al. [62] has found that woman with bothersome tinnitus more often report cardiovascular diseases, thyroid diseases, epilepsy, fibromyalgia, and burnout, whereas in men, bothersome tinnitus is related to alcohol consumption, meniere’s disease, anxiety, and panic disorders. The direction of these relationships needs to be assessed in well-designed prospective studies. Some studies report also differences in therapeutic outcome with women being more responsive than men to some treatments as for instance transcranial magnetic stimulation [63], acoustic stimulation [64], or high definition transcranial direct current stimulation [65]. While sex often has an impact on a primary outcome, it does not mean the outcome is sexually dimorphic (i.e., significantly different between the sexes). Future studies will need to address these aspects, as it may also lead to therapeutic benefits tailored to one or the other of the sexes.

A third limitation is the large heterogeneity in the use of adjustment factors, which may also account for the between-study heterogeneity which was high for factors such as unspecified hearing loss, sensorineural hearing loss, smoking, sex, temporomandibular joint disorder, body mass index, heart failure, migraine, and head injury (*I*^2^ = 69–99%, *p* > 0.05). Quite obviously, meta-analysis would be optimized when pooling studies with compatible statistical measures. Of note, it is interesting to note that there was also a broad heterogeneity in the selection of the models for statistical analyses. Cox regression analyses were used in 8 of 25 studies, three of them with tinnitus being self-reported. Cox regression is traditionally used as a model for when a disease status is achieved, and it also remains (e.g., HIV, mortality, cancer). Whereas in medically assessed tinnitus, there is an increased likelihood of tinnitus being more severe than in self-reported tinnitus; there is a large degree of uncertainty in both cases whether tinnitus was perceived occasionally or constant. Indeed, Edvall et al. [66] recently reported using longitudinal data that the more often occasional tinnitus is perceived, the more likely it will become constant and that constant tinnitus increases the odds of tinnitus becoming permanent. In this study, given the highly dynamic transitions of tinnitus states in individuals with occasional tinnitus, a decision was taken not to use a Cox regression model, and instead rely on generalized estimating equation (GEE) to circumvent these issues (Cederroth, personal communication). This study also reveals that individuals with constant tinnitus differ from those with occasional tinnitus and non-tinnitus controls in that they display increased latencies of Wave V of the auditory brainstem response (ABR). The ABR from individuals with occasional tinnitus was indistinguishable from the non-tinnitus controls. These findings overall suggest, as mentioned above in the case of medically assessed tinnitus, that all methods to ensure that tinnitus was at least chronic and constant may help refine the current picture and reinforce the evidence on reliable risk factors for tinnitus. In this case, then Cox regression models may be justified.

### Future Directions

Prospective cohort studies provide the strongest evidence in risk factor analysis and so there is scope for more of these study designs. Some large population-based prospective cohorts have explored the relationship between tinnitus and various exposures. Conducting new, large, and well-designed prospective cohort studies would be ideal — however, we acknowledge this is a major effort that tinnitus alone may not justify. It would also be worthwhile and resource-friendly to explore the existing databases not only to look for associations between tinnitus and unexplored exposures, but also to validate and replicate previous findings. Establishing a resource with a list of databases including tinnitus phenotypes may be extremely useful to the research community, but the benefits to the society may only derive from high-quality studies. In addition, a broad communication endeavor will be required to improve the phenotypic definitions of tinnitus in existing prospective cohorts such that high-quality data may be acquired in the near future.

## Concluding Remarks

Data identification, synthesis, and reporting have emerged as necessary steps in dissemination and translation of raw data into clinical practice and policy decisions. High-quality primary data acquisition is a pre-requisite for high-quality data synthesis. For exposure assessment, this implies conducting well-designed analytical observational studies to provide stronger evidence. Findings from this review show that tinnitus is related to multiple exposures. However, for most of them, there are insufficient data to conclude a causal relationship. The knowledge of risk factors is crucial for understanding tinnitus etiology as it could also provide insights for pathophysiological mechanisms, contribute to improved tinnitus management, and individualize treatments based on the underlying cause, instead of alleviating the tinnitus percept alone.

## Supplementary Information

Below is the link to the electronic supplementary material.

Supplementary file1 (PDF 262 KB)

Supplementary file1 (PDF 262 KB)

Supplementary file2 (XLSX 232 KB)

## Data Availability

The data extracted for this study can be found in Supplementary Data.
