# Supplementary material for: Low Evidence for Tinnitus Risk Factors: A Systematic Review and Meta-analysis
Source: J Assoc Res Otolaryngol. 2022 Nov 15;24(1):81–94. doi: 10.1007/s10162-022-00874-y (PMC9971395; doi:10.1007/s10162-022-00874-y)

Supplementary Figure 1. Hearing loss (unspecified)

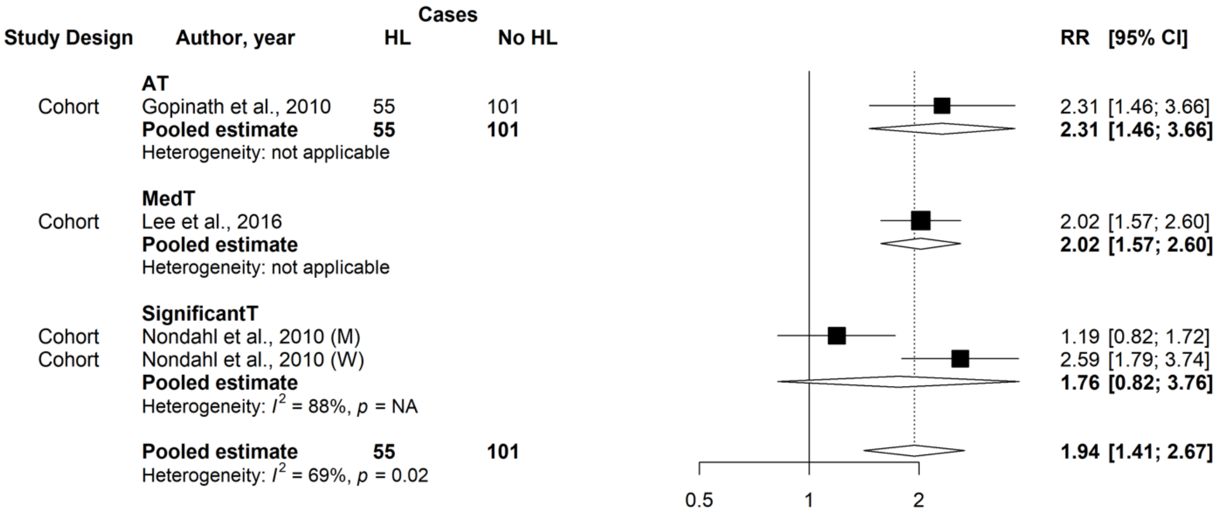

Supplementary Figure 2. Sensorineural hearing loss

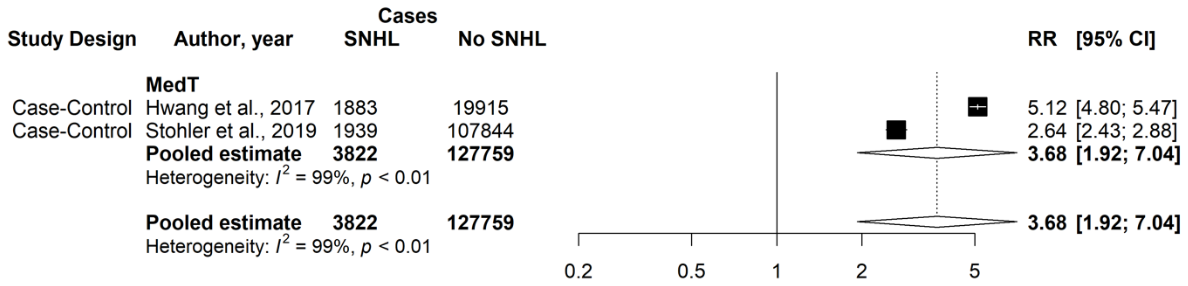

Supplementary Figure 3. Noise exposure (occupational)

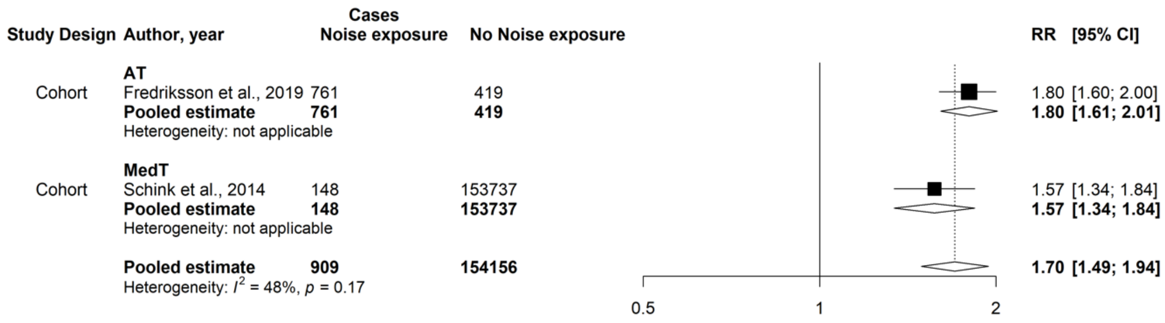

Supplementary Figure 4. Otitis media

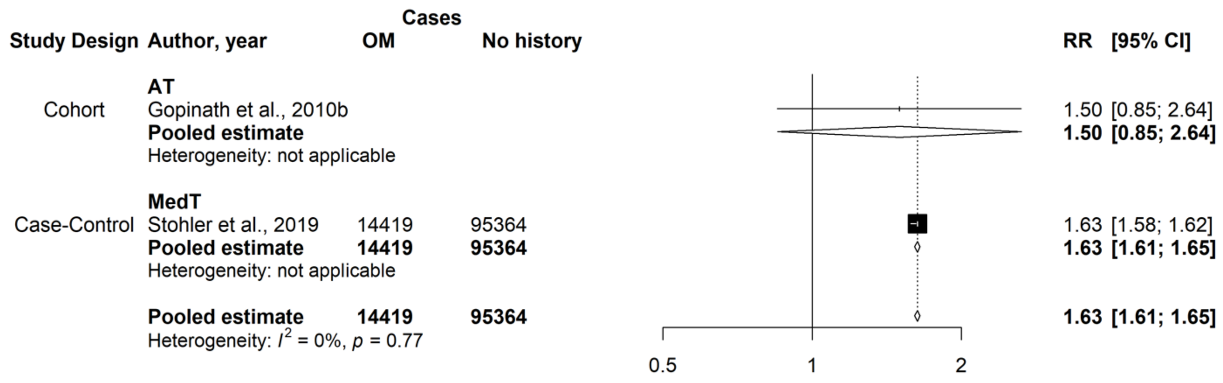

Supplementary Figure 5. Platinum (ototoxic)

Study Design	Author, year	Cases Platinum Therapy	No history	RR [95% CI]
	**AT**			
Cohort	Dille et al., 2011	30	35	5.53 [2.08; 14.68]
	**Pooled estimate**	**30**	**35**	**5.53 [2.08; 14.69]**
	Heterogeneity: not applicable			
	**MedT**			
Cohort(nested case-control)	Whelan et al., 2011			2.80 [1.90; 4.20]
Cohort	Shih et al., 2017	209	964	0.93 [0.10; 1.87]
Cohort	King et al., 2017			2.60 [1.20; 5.50]
	**Pooled estimate**	**209**	**964**	**2.59 [1.83; 3.67]**
	Heterogeneity: I^2 = 1%, p = 0.36			
	**Pooled estimate**	**239**	**999**	**2.81 [1.81; 4.36]**
	Heterogeneity: I^2 = 26%, p = 0.25			

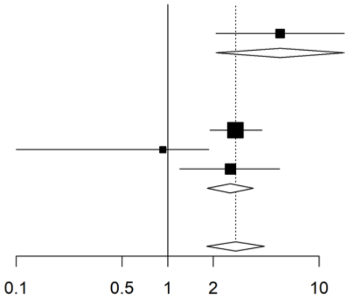

Supplementary Figure 6. Noise exposure (leisure)

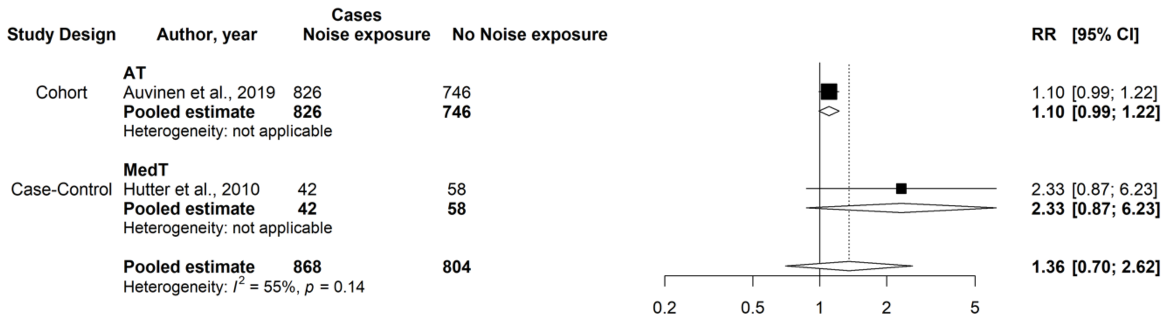

Supplementary Figure 7. Alcohol consumption (high)

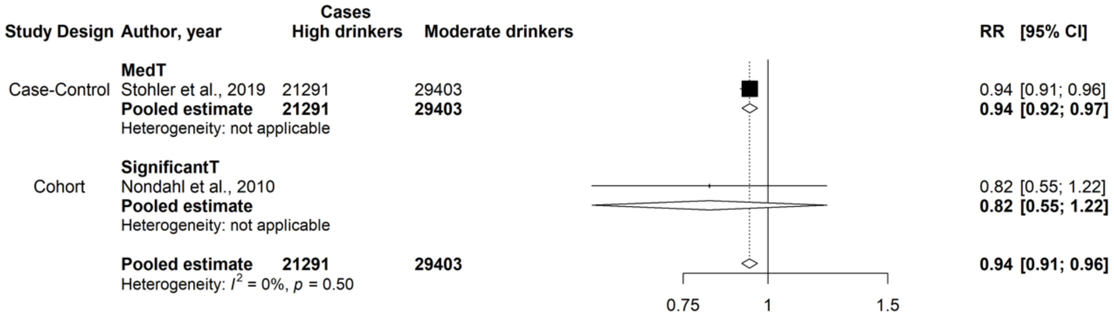

Supplementary Figure 8. Alcohol consumption (low)

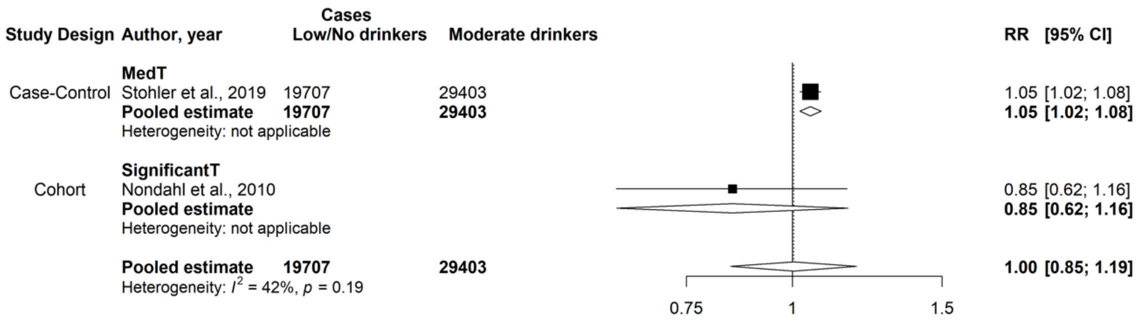

Supplementary Figure 9. Smoking

Study Design	Author, year	Cases Smokers	Non-smokers	RR [95% CI]
	**MedT**			
Case-Control	Stohler et al., 2019	51286	53513	0.98 [0.97; 1.00]
	**Pooled estimate**	**51286**	**53513**	**0.98 [0.97; 1.00]**
	Heterogeneity: not applicable			
	**SignificantT**			
Cohort	Nondahl et al., 2010			1.40 [1.10; 1.79]
	**Pooled estimate**			**1.40 [1.10; 1.79]**
	Heterogeneity: not applicable			
	**Pooled estimate**	**51286**	**53513**	**1.15 [0.81; 1.62]**
	Heterogeneity: $I^2 = 88\%$, $p < 0.01$			

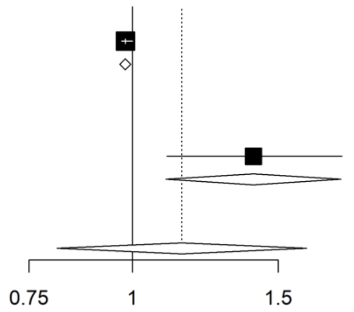

Supplementary Figure 10. Sex/gender

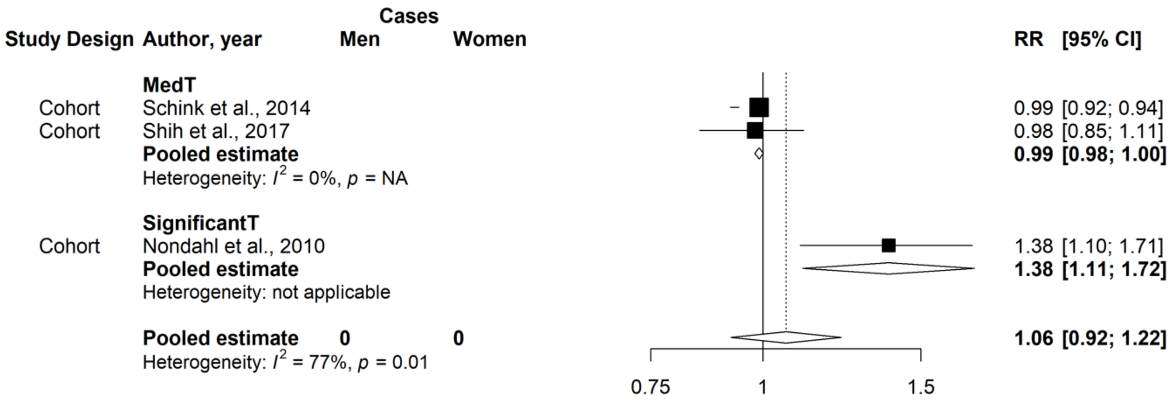

Supplementary Figure 11. Orofacial musculo-skeletal – temporomandibular joint disorder

Study Design	Author, year	Cases TMD	No history	RR	[95% CI]
	**MedT**				
Cohort	Lee et al., 2016	362	530	2.62	[2.29; 3.00]
Case-Control	Stohler et al., 2019	2746	107037	1.64	[1.54; 1.73]
	**Pooled estimate**	**3108**	**107567**	**2.06**	**[1.30; 3.27]**
	Heterogeneity: I^2 = 97%, p < 0.01				
	**Pooled estimate**	**3108**	**107567**	**2.06**	**[1.30; 3.27]**
	Heterogeneity: I^2 = 97%, p < 0.01				

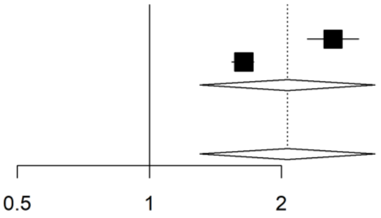

Supplementary Figure 12. Mental health – depression

Study Design	Author, year	Cases Depression	No history	RR	[95% CI]
	**MedT**				
Cohort	Chen et al., 2019	41	265	1.23	[0.80; 1.79]
Case-Control	Stohler et al., 2019	22421	87362	1.31	[1.28; 1.34]
	**Pooled estimate**	**22462**	**87627**	**1.31**	**[1.28; 1.34]**
	Heterogeneity: I^2 = 0%, p = 0.76				
	**Pooled estimate**	**22462**	**87627**	**1.31**	**[1.28; 1.34]**
	Heterogeneity: I^2 = 0%, p = 0.76				

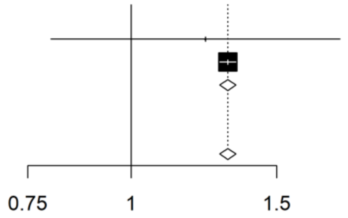

Supplementary Figure 13. Endocrine and metabolic - diabetes

Study Design	Author, year	Cases Diabetes	No history	RR [95% CI]
	**MedT**			
Cohort	Shih et al., 2017	261	912	0.85 [0.71; 0.99]
Case-Control	Stohler et al., 2019	6915	102868	0.85 [0.83; 0.89]
	**Pooled estimate**	**7176**	**103780**	**0.85 [0.82; 0.88]**
	Heterogeneity: I^2 = 0%, p = 1.00			
	**Pooled estimate**	**7176**	**103780**	**0.85 [0.82; 0.88]**
	Heterogeneity: I^2 = 0%, p = 1.00			

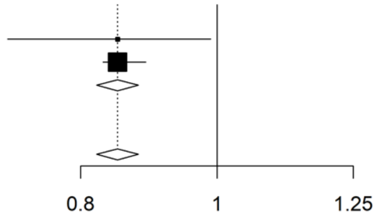

Supplementary Figure 14. Noise exposure (leisure)

Study Design	Author, year	Cases Noise exposure	No Noise exposure	RR [95% CI]
	**AT**			
Cohort	Auvinen et al., 2019	826	746	1.10 [0.99; 1.22]
	**Pooled estimate**	**826**	**746**	**1.10 [0.99; 1.22]**
	Heterogeneity: not applicable			
	**MedT**			
Case-Control	Hutter et al., 2010	42	58	2.33 [0.87; 6.23]
	**Pooled estimate**	**42**	**58**	**2.33 [0.87; 6.23]**
	Heterogeneity: not applicable			
	**Pooled estimate**	**868**	**804**	**1.36 [0.70; 2.62]**
	Heterogeneity: I^2 = 55%, p = 0.14			

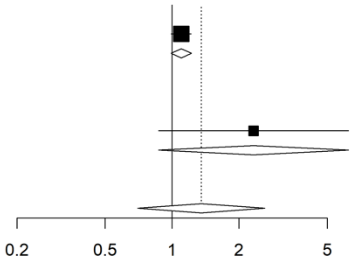

Supplementary Figure 15. Body mass index

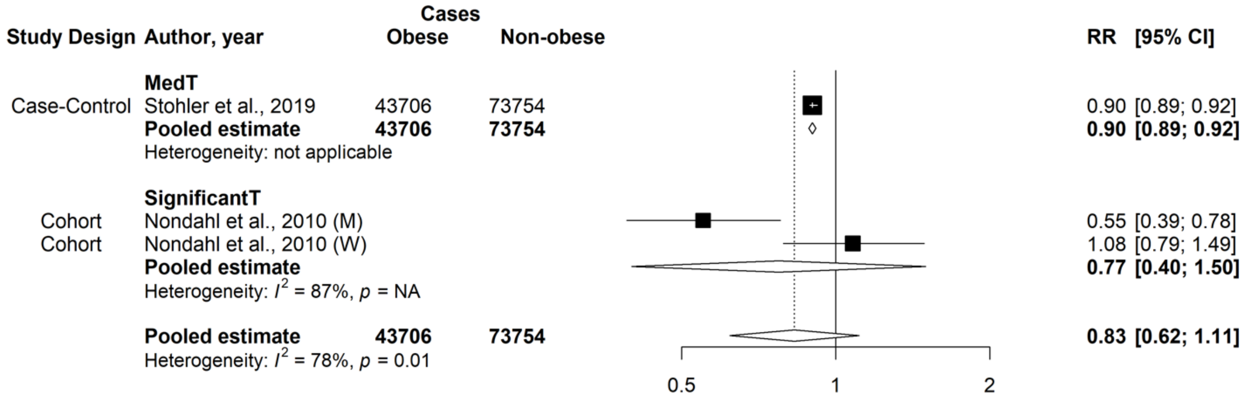

Supplementary Figure 16. Cardiovascular – heart failure

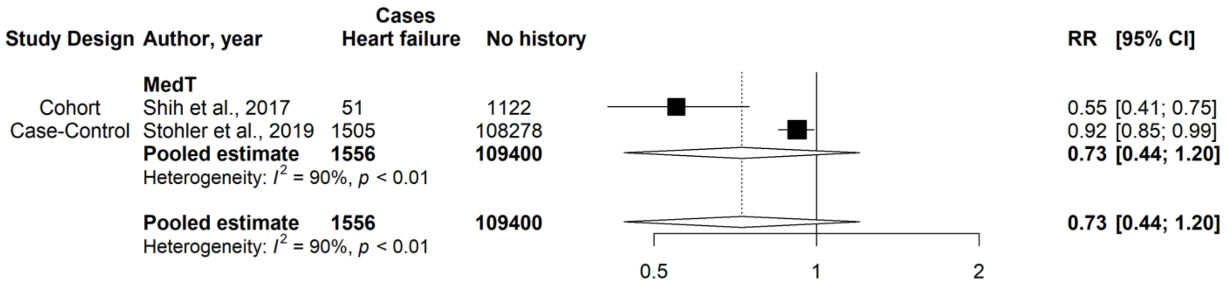

Supplementary Figure 17. Cardiovascular – hypertension

Study Design	Author, year	Cases Hypertensive	Non-hypertensive	RR	[95% CI]
	**AT**				
Cohort	Gopinath et al., 2010b			1.32	[0.85; 2.04]
	**Pooled estimate**			**1.32**	**[0.85; 2.04]**
	Heterogeneity: not applicable				
	**MedT**				
Cohort	Shih et al., 2017	382	791	1.01	[0.81; 1.15]
Case-Control	Stohler et al., 2019	25988	83795	0.98	[0.96; 1.00]
	**Pooled estimate**	**26370**	**84586**	**0.98**	**[0.96; 1.00]**
	Heterogeneity: I^2 = 0%, p = 0.74				
	**Pooled estimate**	**26370**	**84586**	**0.98**	**[0.96; 1.00]**
	Heterogeneity: I^2 = 0%, p = 0.39				

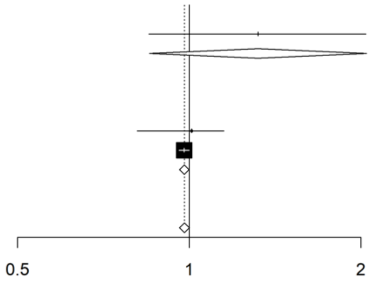

Supplementary Figure 18. Cardiovascular – stroke

Study Design	Author, year	Cases Stroke	No history	RR [95% CI]
	**MedT**			
Cohort	Shih et al., 2017	121	1052	0.98 [0.65; 1.22]
Case-Control	Stohler et al., 2019	3385	106398	0.88 [0.84; 1.93]
	**Pooled estimate**	**3506**	**107450**	**0.94 [0.73; 1.21]**
	Heterogeneity: I^2 = 0%, p = 0.69			
	**Pooled estimate**	**3506**	**107450**	**0.94 [0.73; 1.21]**
	Heterogeneity: I^2 = 0%, p = 0.69			

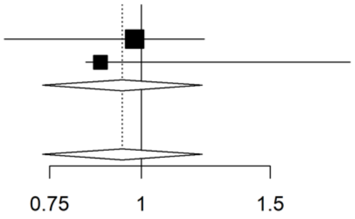

Supplementary Figure 19. Endocrine and metabolic – hyperlipidemia

Study Design	Author, year	Cases Hyperlipidemia	No history	RR [95% CI]
	**MedT**			
Cohort	Chen et al., 2019	30	276	1.46 [0.89; 2.02]
Case-Control	Stohler et al., 2019	12081	97702	1.14 [1.11; 1.18]
	**Pooled estimate**	**12111**	**97978**	**1.18 [1.00; 1.40]**
	Heterogeneity: I^2 = 28%, p = 0.24			
	**Pooled estimate**	**12111**	**97978**	**1.18 [1.00; 1.40]**
	Heterogeneity: I^2 = 28%, p = 0.24			

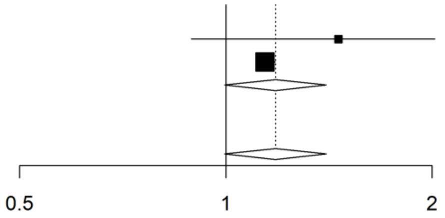

Supplementary Figure 20. Immune-mediated – rheumatoid arthritis

Study Design	Author, year	Cases: RA	Cases: No history	RR [95% CI]
	**MedT**			
Case-Control	Stohler et al., 2019	1479	108304	1.10 [1.02; 1.19]
	**Pooled estimate**	**1479**	**108304**	**1.10 [1.02; 1.19]**
	Heterogeneity: not applicable			
	**SignificantT**			
Cohort	Nondahl et al., 2010 (M)			1.37 [1.08; 1.73]
	**Pooled estimate**			**1.37 [1.08; 1.73]**
	Heterogeneity: not applicable			
	**Pooled estimate**	**1479**	**108304**	**1.19 [0.97; 1.47]**
	Heterogeneity: I^2 = 67%, p = 0.08			

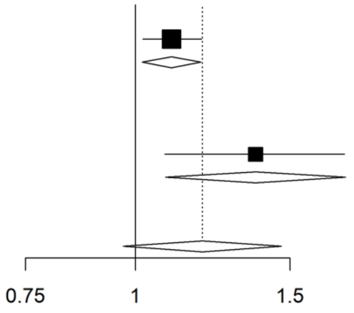

Supplementary Figure 21. Neurological – migraine

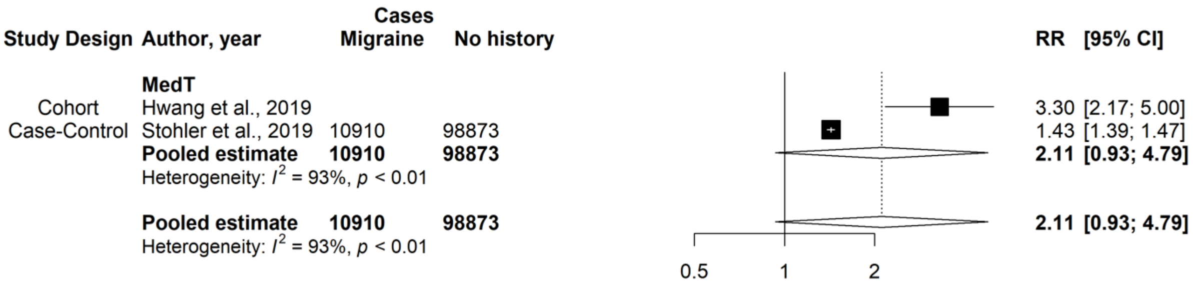

Supplementary Figure 22. Respiratory – Chronic obstructive pulmonary disease

Study Design	Author, year	Cases COPD	Cases No history	RR [95% CI]
	**MedT**			
Cohort	Shih et al., 2017	125	1048	1.00 [0.87; 1.47]
Case-Control	Stohler et al., 2019	2848	106935	0.95 [0.90; 1.00]
	**Pooled estimate**	**2973**	**107983**	**0.95 [0.90; 1.00]**
	Heterogeneity: I^2 = 0%, p = 0.71			
	**Pooled estimate**	**2973**	**107983**	**0.95 [0.90; 1.00]**
	Heterogeneity: I^2 = 0%, p = 0.71			

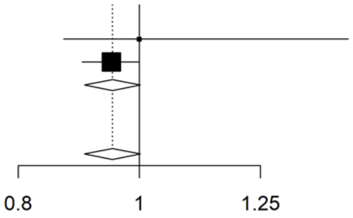

Supplementary Figure 23. Traumatic head and neck injury – head injury

Study Design	Author, year	Cases Head Injury	No history	RR [95% CI]
	**MedT**			
Cohort	Dougherty et al., 2013	122	112	1.17 [0.88; 1.56]
Cohort	Shih et al., 2017	104	1069	0.97 [0.90; 0.98]
Case-Control	Stohler et al., 2019	603	109180	1.17 [1.04; 1.32]
	**Pooled estimate**	**829**	**110361**	**1.08 [0.92; 1.26]**
	Heterogeneity: $I^2 = 79\%$, p = NA			
	**Significant T**			
Cohort	Nondahl et al., 2010			1.76 [1.40; 2.22]
	**Pooled estimate**			**1.76 [1.40; 2.22]**
	Heterogeneity: not applicable			
	**Pooled estimate**	**829**	**110361**	**1.21 [0.97; 1.52]**
	Heterogeneity: $I^2 = 91\%$, $p < 0.01$			

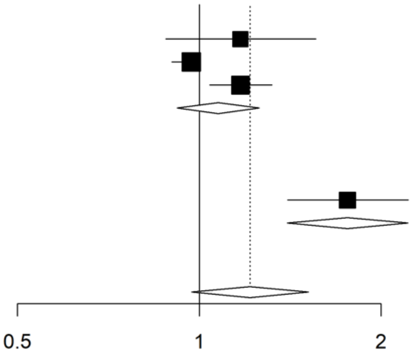

Supplementary Figure 24. Traumatic head and neck injury – whiplash

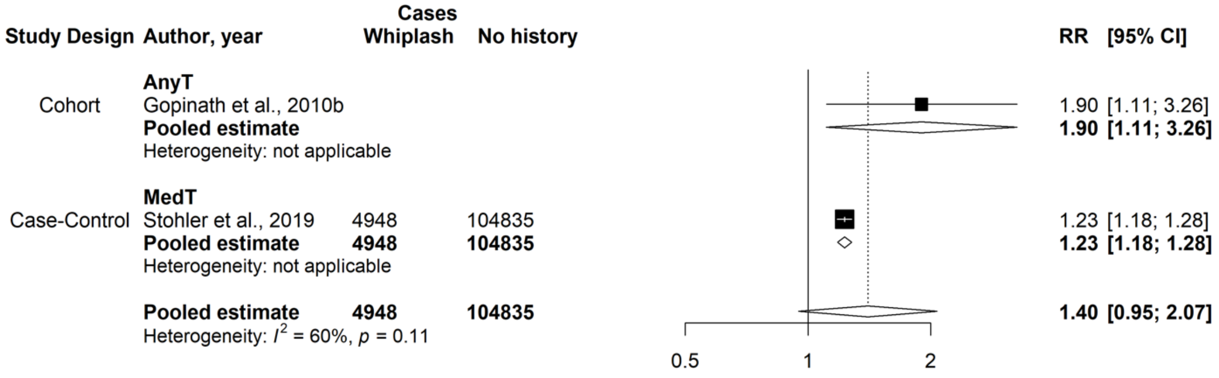

Supplement: Supplementary file 1 — Supplementary file1 (PDF 262 KB) [file 10162_2022_874_MOESM1_ESM.pdf]
